# The Analysis of Key Factors Related to ADCs Structural Design

**DOI:** 10.3389/fphar.2019.00373

**Published:** 2019-04-24

**Authors:** Haichao Tang, Yan Liu, Zhaojin Yu, Mingli Sun, Lu Lin, Wensi Liu, Qiang Han, Minjie Wei, Ying Jin

**Affiliations:** ^1^Department of Pharmacology, School of Pharmacy, China Medical University, Shenyang, China; ^2^Liaoning Engineering Technology Research Center for the Research, Development and Industrialization of Innovative Peptide Drugs, China Medical University, Shenyang, China; ^3^Liaoning Research Institute of Family Planning, Shenyang, China

**Keywords:** antibody–drug conjugates, precision choice antibody and antigen, elaborate modification linkers, proper payloads, optimized linker-payload linkage

## Abstract

Antibody–drug conjugates (ADCs) have developed rapidly in recent decades. However, it is complicated to map out a perfect ADC that requires optimization of multiple parameters including antigens, antibodies, linkers, payloads, and the payload-linker linkage. The therapeutic targets of the ADCs are expected to express only on the surface of the corresponding target tumor cells. On the contrary, many antigens usually express on normal tissues to some extent, which could disturb the specificity of ADCs and limit their clinical application, not to mention the antibody is also difficult to choose. It requires to not only target and have affinity with the corresponding antigen, but it also needs to have a linkage site with the linker to load the payloads. In addition, the linker and payload are indispensable in the efficacy of ADCs. The linker is required to stabilize the ADC in the circulatory system and is brittle to release free payload while the antibody combines with antigen. Also, it is a premise that the dose of ADCs will not kill normal tissues and the released payloads are able to fulfill the killing potency in tumor cells at the same time. In this review, we mainly focus on the latest development of key factors affecting ADCs progress, including the selection of antibodies and antigens, the optimization of payload, the modification of linker, payload-linker linkage, and some other relevant parameters of ADCs.

## Introduction

In traditional tumor treatment, chemotherapy is one of the main treatment strategies. However, the toxicity from non-specific accumulation in normal tissues, narrow therapeutic window and low tolerance all limit chemotherapy drug development in the tumor treatment process (Atkins and Gershell, 2002; Alley et al., 2010; Ashley et al., 2011). In recent decades, scientists have gained an in-depth understanding of cancer biology, taking advantage of some unique features of tumor cells to transform cancer treatment from previous chemotherapy drugs to tumor-targeted therapies. Monoclonal antibodies and polypeptides which bind to specific markers on the tumor cell’s surface provide targeted therapeutic approaches and are both less toxic. However, whether they are monoclonal antibodies or peptides, they both lack potency in killing tumor cells.

**FIGURE 1:**
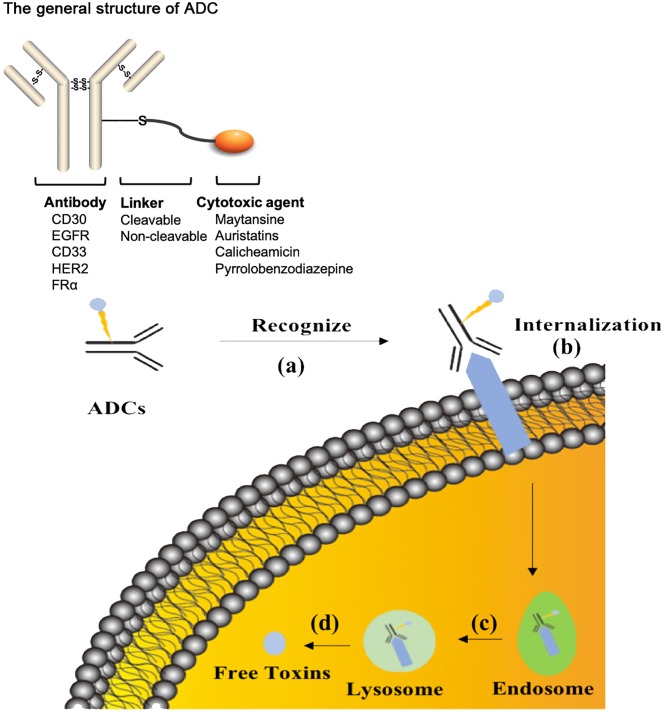
The process of ADCs exerting activity. **(a)** ADCs specifically recognize cancer-associated antigens in the blood system. **(b)** ADCs are internalized into tumor cells during the formation of antibody-antigen complex. **(c)** ADCs are normally transported to lysosome from endosome. **(d)** The linker or antibody are broken in the lysosome conditions to release free toxins. ADCs, antibody drug conjugates.

**FIGURE 2:**
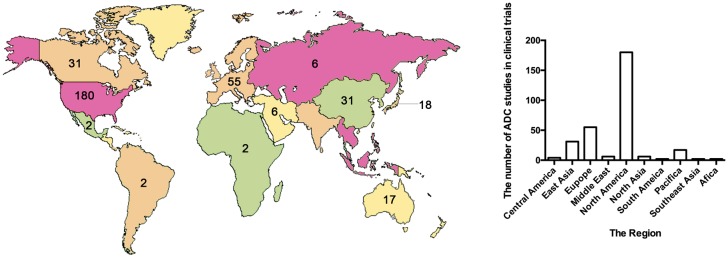
The map and statistical graph depict regions where developed antibody–drug conjugates. The numbers in the figure indicate the amounts of ADCs in the clinical phase of the region. The data comes from ClinicalTrials.gov.

**FIGURE 3:**
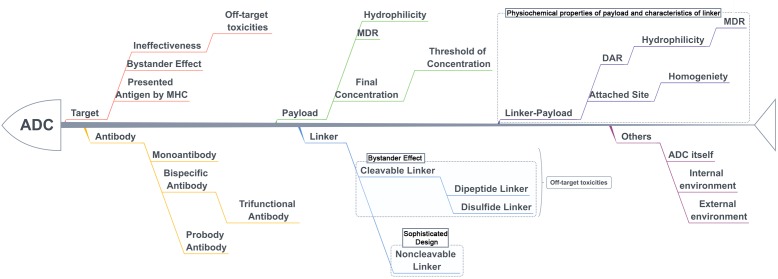
The key parameters associated with efficacy and toxicities with ADCs.

The treatment strategy of antibodies armed with toxins to selectively kill target cells was first proposed in 1970 (Moolten and Cooperband, 1970). The tumor-targeting drug conjugates integrate targeted biomolecules with therapeutic small molecule toxins to specifically recognize the tumor tissues and kill the tumor cells, thereby improving the therapeutic index of the toxins and the insufficient efficacy of antibodies or peptides. The tumor-targeting drug conjugates mainly compose of ADCs that generally couple antibodies which specifically recognize the surface antigens of tumor cells with chemical toxins which effectively kill tumor tissues through linkers, and ADCs exert killing activity by bringing the chemical toxins into the tumor cells. In general, the antibody specifically binds to the tumor cell surface antigen, and the antigen mediates the endocytosis of the ADC and then releases free toxins (Figure 1), but the downsides are that immunogenicity, poor internalization and the instability of the linker give rise to insecurity and ineffectiveness (Chari, 2008). More than 60 ADCs have been in the process of clinical development until 2016 (Carter and Lazar, 2017), there are almost 204 ADCs (Supplementary Table S1) that aim for cancer in clinical development by 2018, including at least nine of which have entered phases III and IV clinical trials^1^. It indicates that ADCs are coming to the center-stage of research field in recent years especially in North America (Figure 2). However, until today, only ado-trastuzumab emtansine (T-DM1, Kadcyla^@^) and brentuximab vedotin (Adcetris^@^) are approved by FDA and on the market (Mullard, 2013; Thomas et al., 2016). There are many reasons for the dilemma, including the complexity of the composition of the ADC itself, and the fact that the tumor microenvironment or physiological conditions in animals are different from the human so that the evaluation of ADC efficacy by animal models is not applicable to humans. Beck et al. (2017) published a review paper about the strategies and challenges for the next generation of ADCs in 2017. However, ADCs are developing rapidly and some novel technologies may bring new considerations. Thus, this review mainly focuses on imperative factors that are associated with ADC efficacy (Figure 3).

## The Selection of Antibody and Antigen

Normally, the antigen specific to the cancer cell should be a priority after determining the indications for ADC. Ideally, the antigen should express highly and homogeneously on the surface of the cancer cells (Sievers and Senter, 2013; Damelin et al., 2015). When the antibody combines with the antigen specifically, the antibody-antigen complex should be internalized by antigen-mediated endocytosis, and then the free payloads are released through lysosomal trafficking. As a result, the payloads are concentrated in cancer cells and exert the cytotoxic effect (Erickson et al., 2006). Currently, the predominant therapeutic limitations are the ineffectiveness and the off-target toxicities of ADCs, which are caused by the finite internalization and the low expression of antigens to some extent. Therefore, some researchers came up with some approaches to counteract these problems such as utilizing the anti-tumor angiogenesis antibody, non-internalizing ADC, or bispecific antibody.

### The Utilization of Anti-tumor Angiogenesis Antibody

Some researchers proposed a strategy that using an anti-tumor angiogenesis ADC to selectively kill cancer cells due to the process without the involvement of internalization, which could improve the deficient efficacy caused by finite internalization. For example, Palumbo et al. (2011) reported that the ADC composes of an anti-angiogenesis LC19 antibody to selectively target to the tumor blood vessels, the strategy showed a long-term anti-tumor effect. However, ADCs of the anti-tumor vessel may elicit off-target toxicities to normal tissues due to non-specificity of antigen expression and resistance of vessel co-option in some particular tumor tissues (Kuczynski et al., 2016). This requires choosing antibodies based on proper antigens. Seaman et al. (2017) applied the anti-CD276 antibody to the ADC to improve the non-specificity. The CD276 expresses in angiogenic tumor vessel, existed vasculature and tumor cells. Moreover, the anti-CD276 antibody is capable of identifying the normal and pathological angiogenesis. The anti-CD276 ADC evaded the vessel co-option and displayed a dual-targeting ability thus displaying effective anti-tumor activity (Seaman et al., 2017).

### Preparing Non-internalizing ADCs

An approach to prepare non-internalizing ADCs to target corresponding antigens needed to be developed. For instance, the ADC took advantage of a diabody without an Fc region to target the matching antigen and an additional chemical activator to cut the linker, and then release the free payload to penetrate into tumor cells (Rossin et al., 2018). This strategy is able to increase the anti-tumor activity and avoid some factors can sacrifice the efficacy of ADCs such as interstitial pressure and epithelial barriers from the tumor cells.

### The Selection of the Bispecific Antibody

On one hand, in terms of the deficiency of internalization, Li et al. (2016) used a bispecific antibody to target two non-overlapping epitopes of one antigen, which increased the affinity between antibody and antigen. For example, an anti-HER2 biparatopic antibody displayed better internalization, lysosomal trafficking, and degradation of the antibody-antigen complex relative to the traditional T-DM1 (Li et al., 2016). However, the superior affinity also may trigger a controversy about whether the biparatopic ADC would induce on-target toxicities to healthy tissues. Though this study also further indicated that the biparatopic ADC has an acceptable safety profile due to the threshold of antigen. It is unable to form the antibody-antigen complex if the expression level of antigen below the threshold. However, it seems arduous to avoid the problem due to the uncertain threshold. Theoretically, a higher affinity antigen–antibody could make more ADC molecules combine with tumor cells thus having more accumulation, but a lower affinity may allow ADCs to penetrate into tumor cells more effectively. Scientists are still looking for antigen–antibody with proper affinity (Rudnick et al., 2011). It needs further research (Tsumura et al., 2018).

Also, some researchers proposed to use the probody of antibody to solve the on-target toxicities, which may also be applied to ADCs. This strategy used masking peptide to cover up the active sites of the antibody then hydrolysis of the shelter to expose the antibody to target cancer tissues to exert activity (Desnoyers et al., 2013), which allays the indistinct recognition of ADCs in the blood circulation.

On the other hand, the bispecific antibody is able to selectively bind two distinct antigens on a cancer cell to avoid the off-target toxicities. For example, the bispecific antibody simultaneously targets the HER2 and PRLR double positive (HER2^+^/PRLR^+^) breast cancer cells to enhance the internalization and activity of the ADC, and to decrease the off-target toxicities to the healthy cells (Andreev et al., 2017). Nevertheless, targeting double-antigens is ineligible for most heterogeneous tumor cells, since it may trigger their escape mechanism. Furthermore, the bispecific antibody could be used to target the immunosuppressive molecule and tumor-specific antigen on the tumor cells simultaneously to improve the efficacy of ADC. The ADC targeting CD47 that an immunosuppressive receptor and TAA double positive (CD47^+^/TAA^+^) tumor cells could block the immunosuppression to augment the killing activity of the ADC (Dheilly et al., 2017). Currently, there are more than 70 bispecific antibodies applied in clinical trials (see text footnote 1), two of them have used on the market. These specific antibodies seem to change some imperfect phenomena of ADCs (Piccione et al., 2015). Moreover, the trifunctional antibody also could be used to ADC (Krishnamurthy and Jimeno, 2018), which possess an arm to target the tumor cells, the second is used to target T cells, the remaining Fc region to recruit some immune cells. Using the trifunctional antibody to link a small molecule toxin seems to improve the deficient specificity and the killing potency of ADCs. Though the bispecific or trifunctional prospect is promising to improve potency and specificity to increase market competitiveness of ADCs, the challenge of determining the target combination still remains.

### The Bystander Effect to Heterogeneous Tumors

Some reports also demonstrated that some ADCs may take advantage of the physical and chemical properties of linkers and the microenvironment of the tumor to release free payloads to kill those adjacent negative-antigen cancer cells. The process is the bystander effect (Kovtun et al., 2006; Okeley et al., 2010). ADC was metabolized to release uncharged and membrane-permeable toxic metabolites after being internalized in positive-antigen cancer, which is able to kill adjacent antigen-negative cancer cells by membrane-penetration (Kellogg et al., 2011). This has a great significance for some heterogeneous tumor cells. Admittedly this was that the bystander effect may also cause non-specific killing of normal cells. Therefore, it requires to have rational selection and design of payloads and linkers based on the target to avoid the adverse effects from bystander effect.

### The Selection of Antibody Isotype

Within IgG isotypes, IgG1, IgG2, IgG4 have been used to develop therapeutics, but IgG3 isotypes are not used as therapeutics owing to a significantly faster clearance rate (Jefferis, 2007). Further, most ADCs use IgG1 isotype currently (Beck et al., 2017). IgG1 isotype may exert ADCC (antibody-dependent cell-mediated cytotoxicity) and CDC (complement-dependent cytotoxicity) to improve ADCs activity further, whereas IgG2 and IgG4 are typically deficient in their effector functions (Salfeld, 2007). However, the PD-1 antibodies (Nivolumab and Pembrolizumab) used IgG4 isotype, which may be due to the PD-1 antibody only needing to block the interaction between PD-1 and PD-L1 to increase immune system function to produce anti-tumor activity, which is needed to avoid the toxicity to T cells from ADCC and CDC. Therefore, the choice of isotype also needs careful consideration.

### The Consideration of Antigen

Glycosylation of antigen also could affect the design of ADCs. On the one hand, if glycosylated antigen specifically exists on the tumor cell surface, it will have an important implication to be a target of an ADC. For example, a monoclonal antibody targeting glycosylated PD-L1 (gPD-L1) to disrupt PD-L1/PD-1 interaction. The gPD-L1 is mainly expressed on tumor tissues, which improves non-specific expression of PD-L1 in some immune cells to limit toxicity (Li et al., 2018). On the other hand, the steric structure of a glycosylated antigen plays a certain protective role, which will block the interaction between the antibody and specific sites on the antigen surface. Therefore, we need to have a more comprehensible understanding on designing ADCs.

**FIGURE 4:**
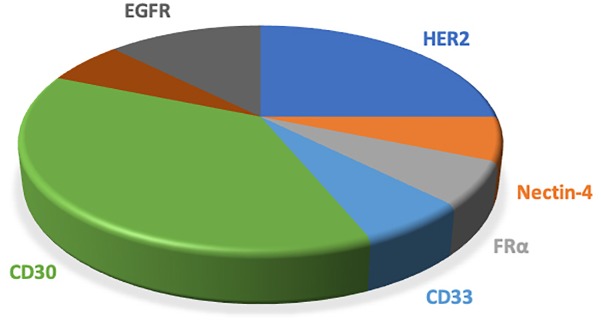
The pie chart shows the antigens applied to the clinical phase III/IV trials of ADCs. ADCs, antibody drug conjugates; MDR, multiple drug resistance; DAR, drug-to-antibody ratio.

Most ADCs utilize the antigens on the tumor cells surface, which are limited in their specificity relative to intracellular antigens. Taking advantage of the antigen presentation feature of MHC that caused tumor-specific endogenous antigen expression on the cell surface overcomes the inaccessibility of intracellular antigens. Further, the MHC-I/peptide complex is recognized by the ADCs that mimic the characterization of TCR, which will produce superior specificity and potency (Lai et al., 2018). Table 1 and Figure 4 showed the antigens used in phase III/IV trial currently (see text footnote 1).

**Table 1:** Current clinical phase III/IV trials of ADCs.

NCT number	Name	Conditions	Payloads	Target
NCT03523585	DS-8201a	Breast cancer	Topoisomerase I inhibitor	HER2
NCT03734029	DS-8201a	Breast cancer	topoisomerase I inhibitor	HER2
NCT03529110	DS-8201a	Breast cancer	topoisomerase I inhibitor	HER2
NCT03262935	SYD985	Metastatic breast cancer	DUBA	HER2
NCT03474107	Enfortumab vedotin	Ureteral cancer| urothelial cancer| bladder cancer	MMAE	Nectin-4
NCT02631876	Mirvetuximab soravtansine (IMGN853)	Epithelial ovarian cancer| primary peritoneal carcinoma| fallopian tube cancer| ovarian cancer	DM4	FRα
NCT02785900	Vadastuximab talirine (SGN-CD33A; 33A)	Acute myeloid leukemia	PBD	CD33
NCT01990534	Brentuximab Vedotin	Hodgkin lymphoma	MMAE	CD30
NCT03677596	Inotuzumab ozogamicin	Leukemia| precursor b-cell lymphoblastic leukemia-lymphoma| acute lymphoblastic leukemia	Calicheamicins	CD22
NCT02573324	Depatuxizumab mafodotin (ABT-414)	Glioblastoma	MMAF	EGFR
NCT01100502	Brentuximab vedotin (SGN-35)	Disease, Hodgkin	MMAE	CD30
NCT01777152	Brentuximab vedotin	Anaplastic large-cell lymphoma| non-Hodgkin lymphoma| T-cell lymphoma	MMAE	CD30
NCT01909934	Brentuximab vedotin	Anaplastic large-cell lymphoma	MMAE	CD30
NCT03419403	Depatuxizumab mafodotin (ABT-414)	Glioblastoma multiforme	MMAF	EGFR
NCT01712490	Brentuximab vedotin	Hodgkin lymphoma	MMAE	CD30
NCT02166463	Brentuximab vedotin	Hodgkin lymphoma	MMAE	CD30

*DUBA, duocarmycin-hydroxybenzamide-azaindole; MMAE, monomethyl auristatin E; MMAF, monomethyl auristatin F; DM4, maytansine 4; PBD, pyrrolobenzodiazepine dimers.*

## The Selection of Payloads

Once the target is determined, the proper choice of payload becomes a critical part of ADC. The final potency of ADCs mainly depends on the concentration of payloads in tumor cells; thus some researchers are dedicated on improving the DAR of ADCs to increase the accumulation of drugs in tumor cells. Zhang et al. (2018b) indicated that activity of the ADC still remains constant though augmenting the payload concentration, and this also could magnify the toxicity to normal tissues at the same time. This suggests that the threshold of payload concentration also needs consideration. In summary, choosing the applicable payloads and designing the appropriate DAR is important for the final concentration of the payloads in tumor cells.

### Improving the Efficacy of ADCs

Early ADCs used drugs that have been approved for clinical use such as vinblastine and doxorubicin, but the low clinical activity of these drugs resulted in suboptimal ADCs efficiency. Some cytotoxins were too toxic to be non-target agents in clinical application, but they seemed to be more promising as payloads for ADCs. At present, the dose of the payloads in tumor cells is required to be the picomolar range to kill these cancer cells (Chari et al., 2014). Also, payloads must possess acceptable solubility and decent sites to react with linkers. These all limited the selection of payloads. Currently, most payloads are derivatives of the microtubule inhibitor family, such as the auristatin and maytansine (Beck and Reichert, 2014). Brentuximab vedotin (Adcetris^@^), approved by FDA in 2011, composes of MMAE and cAC10 mAb (chimeric IgG1 antibody) via a protease-cleavable dipeptide linker to target tumor antigen CD30 (also known as TNFRSF8) for the treatment of Hodgkin’s lymphoma and ALCL (ki-1 lymphoma) (Senter and Sievers, 2012; Younes et al., 2012). Ado-trastuzumab emtansine (Kadcyla^@^), approved in 2013, consists of a stable thioether linker (SMCC) attached to trastuzumab (anti-human epidermal growth factor receptor-2 antibody, anti-HER2 antibody) and DM1 (maytansine derivative) for the treatment of advanced breast cancer (Lambert and Chari, 2014). Both adopted the microtubule inhibitor family as payloads, yet auristatins and maytansines are only able to exert activity in cell proliferation and they are hydrophobic, which will disturb their activity. Thus, some novel payloads or the original payload structural modifications such as the improvement of hydrophilicity will become the hotspots of the future payload research (Burke et al., 2017). At present, some novel ADCs have better activity and have been through clinical phase III/IV (Table 1).

The first commercially available ADC was gemtuzumab ozogamicin (GO) that consists of calicheamicins which damage DNA (Walker et al., 1992) for the treatment of AML. However, GO showed no significant improvement in overall survival (OS) compared with the calicheamicin agent alone, and had a higher mortality rate and was recalled in 2010 (Petersdorf et al., 2013; Kharfan-Dabaja, 2014). This is because calicheamicin is hydrophobic in that almost only 50% could be conjugated, and only approximately 50% of free drugs are eventually released in the conjugated drugs (Beck et al., 2010; Senter and Sievers, 2012), resulting in a significant decrease in potency. To overcome these limitations, some novel targeted DNA agents have been broadly developed. Pyrrolobenzodiazepine dimers (PBDs) have already become a new choice, it may attach to the linker that conjugated to the antibody, and has the ability to overcome MDR relative to the commonly used calicheamicin as a substrate of *P*-glycoprotein (Kung Sutherland et al., 2013; Stein et al., 2018). The IMGN779 (NCT02674763) utilized DGN462 that a novel drug with DNA-alkylating activity also demonstrated better anti-tumor activity and tolerability (Kovtun et al., 2018).

### Avoiding MDR

The MDR has always been a barrier and one of the important factors affecting the therapeutic effect in the cancer treatment. The MDR is still an impeditive factor of using ADCs. This is because the essence of ADC’s activity is that the payloads in tumor cells exert cytotoxicity, and these payloads may be affected by MDR. Many studies concentrate on the modification of drug-linker that, by increasing hydrophilicity, circumvents MDR caused by the overexpression of efflux pumps because the substrates of MDR1 were hydrophobic in general. Moreover, some novel payloads such as PBD, DGN462, and tubulysins cooperate with ADCs to display better anti-tumor activity in MDR^+^ tumor cells (Burke et al., 2018; Kovtun et al., 2018; Stein et al., 2018). ADCs are susceptible to hydrophobicity to be insensible to MDR^+^ cells, thereby it is essential to improve the hydrophilicity to escape from MDR to increase the activity of ADCs (Kovtun et al., 2010).

## The Modification of Linker

Although the linker may be not directly correlated with the final potency of ADC (Lee et al., 2018b), the potency of ADC is dictated by the concentration of payload accumulated in tumor cells, and the payload release is determined by the stability of the linker. Thus, the linker is crucial for a perfect ADC, and it determines the stability, efficacy, and even the ability to overcome MDR. The basic requirement of the linker is to make the payload attach to the antibody, stabilize the payload in the circulation system, and is labile to release the free payload into cancer cells when the antigen–antibody complex is formed (Doronina et al., 2006). Currently, linkers are mainly divided into the cleavable linkers and the non-cleavable linkers.

### The Comparison of Cleavable Linkers With Non-cleavable Linkers

The cleavable linkers normally take advantage of the difference of tumor microenvironment and normal physiological environment to release the payloads that may be membrane-permeable and can produce the bystander effect. The non-cleavable linkers need to meet the requirement that the antibody and linker must be disconnected after the formation of the antigen–antibody complex enter the lysosomal trafficking. This may cause the bystander effect that is a passive transport process to weaken, caused by the membrane-impermeability of linker-payloads connected with polar amino acids. Both types of linkers have their advantages and disadvantages, which are applied to the clinical trials (Chari et al., 2014; Bialucha et al., 2017). However, about 2/3 ADCs used cleavable linkers in the current clinical trials (Lambert and Berkenblit, 2018), in which mainly are dipeptide linkers and disulfide linkers. The non-cleavable linkers are not only more stable to escape from the off-target toxicities than cleavable linkers (Lu et al., 2016), but also may overcome the barrier of multiple-drug resistance (MDR) (Shefet-Carasso and Benhar, 2015; Beck et al., 2017; Nasiri et al., 2018) for the reason that the payload connected with polar amino cannot be a substrate of MDR1, which will improve the MDR phenomenon. However, the non-cleavable linkers need a more elaborate process to produce activity such as the internalization and metabolism of the antibody in the lysosome, which is a prerequisite to release active payloads to exert killing activity (Rosenberg, 2006; Lambert and Berkenblit, 2018), and the polar amino-linker-payload also needs a distinct transporter to carry it from the lysosome to cytoplasm to work (Hamblett et al., 2015; Kinneer et al., 2018; Lee et al., 2018b), which makes the design of ADC more complex to limit the utilization of non-cleavable linkers. The cleavable linkers are more vulnerable to lead to off-target toxicities, but the process of exerting effects is more comprehensible thus researchers are dedicated to modifying the cleavable linkers to overcome their weakness and to increase their stability in the circulation (Sanderson et al., 2005; Kellogg et al., 2011).

### The Analysis of Cleavable Linkers

The cleavable linker can metabolize some cell-permeable metabolites to exert the bystander killing effect. The cleavable sulfo-SPDB-DM4 linker produced cell-permeable catabolites to display a better activity than non-cleavable SMCC-DM1 linker (Bialucha et al., 2017). Also, the application of the sulfonate group improved hydrophilicity to increase the exposure of ADC to the antigen to promote killing activity. The brentuximab vedotin (SGN-35) took advantage of a cleavable dipeptide linker to release free MMAE, and the MMAE may permeate adjacent cells to exert killing activity which is important to some heterogeneous tumor cells. Moreover, the dipeptide linker offers ADC better stability in the circulation, and is more specific to tumor cells (Katz et al., 2011). The protease cleavage pathway is not restricted to cathepsin B, various cysteine cathepsins can cleavage the dipeptide linker, such as cysteine cathepsins B, K, L, and S. It seems to explain why the dipeptide linkers cannot be insensitive to tumors, caused by the insufficient expression of protease (Caculitan et al., 2017), which is one of the reasons why some protease-sensitive linkers are widely used by ADCs.

In particular, the design of the valine-citrulline (val-cit) linker, the most frequent in dipeptide linkers, needs to consider the connection to the phenol-containing payloads; diverse electron groups affect the degrees of immolation of the linker to influence the different potency of an ADC (Zhang et al., 2018a). However, the val-cit dipeptide linker is not conducive to preclinical research to appraise the efficacy of ADCs due to instability in mice (Dokter et al., 2014). Anami et al. (2018) reported a glutamic acid-val-cit linker replaced val-cit dipeptide linker, which could alleviate the flaw of instability in the mice plasma and retain the cathepsin-mediated cleavage mechanisms, thus boosting preclinical application of some ADCs. The acidic tripeptide linker could increase the polarity of ADCs to improve solubility to increase the therapeutic potency (Anami et al., 2018). However, one of the studies suggests that activity of the ADC with cleavable valine-citrulline-p-aminobenzyl-carbamate-monomethyl auristatin F (val-cit-PABC-MMAF) is much less than the ADC with non-cleavable maleimidocaproyl-MMAF (Doronina et al., 2006), which may be due to the character of payloads rather than the linker. The metabolites of some payloads are more effective than the prototypes. The non-cleavable linkers are not widely applied to ADCs since many payload derivatives attached to an amino cannot satisfy the killing potency of ADCs.

The disulfide linker utilized the difference of glutathione (GSH) levels between the tumor microenvironment and the physiological environment of normal tissues to produce activity (Meister and Anderson, 1983; Dubikovskaya et al., 2008), which is more labile in tumoral hypoxia conditions (de Groot et al., 2001). At present, the main obstacle of the disulfide linker is the instability, which is mainly improved by increasing steric hindrance to relieve the vulnerability. ADCs using the disulfide linkers have inferior potency *in vivo* due to the more rapid clearance of payloads compared with the non-cleavable thioether linkers that displayed more potent activity (Lewis Phillips et al., 2008). The trastuzumab emtansine (T-DM1) consists of non-cleavable thioether linker and a maytansine derivate, which has better anti-breast cancer activity. The linker contained a cyclohexane carboxylate and a maleimidomethyl group. The ionized metabolite cannot kill surrounding normal cells due to its impermeability after ADC metabolized, thus the ADC has a better safety (LoRusso et al., 2011). The non-cleavable linkers are stricter in the choice of antigens compared with cleavable linkers, yet fewer toxicities (Polson et al., 2009). Zhang et al. (2016) reported that using methy- and cyclobutyl-substituted disulfide with efficient immolation demonstrated more potent killing activity than cyclopropyl-substituted disulfide with non-immolation. Also, this reflects that the immolation of the linker is imperative to the potency of ADC (Zhang et al., 2016). However, the anti-tumor activity is more determined by the cleavage of the linkers only when payloads require complete cleaving to exert activity (Caculitan et al., 2017). Thus, new research could focus on developing payloads that do not require the production of pharmacological effects with prototype drugs. Also, future studies could focus on developing some novel technologies of payload-linker to improve the activity of ADCs such as SYD985 based on a cleavable linker-duocarmycin payload (NCT03262935) (Dokter et al., 2014).

## The Payload-Linker Linkage

With the development of ADCs, the drug-linker linkage that goes hand in hand with the efficacy of ADCs is more critical (Nasiri et al., 2018). In order to give full play to ADCs’ activity in tumor cells, it is necessary to effectively design the payload-linker according to the physicochemical properties of the payloads and the characteristics of the linkers.

### The Consideration of the Sites of Payload-Linker

The sites of the payload-linker are essential conditions to consider due to the attaching-sites being correlated with homogeneity that is related to the therapeutic index. In the early stages of ADCs development, the lysine on the antibody was used as the site to attach the linker, which caused great heterogeneity. Later, Adcetris^@^ used the cysteine that only eight free cysteines per antibody to link through disulfide bonds, which reduced the ADCs heterogeneity. In recent years, to ensure ADC homogeneity, researchers have developed some site-specific methods, such as THIOMAB (Junutula et al., 2008; Chudasama et al., 2016).

### The Modification of Payload-Linker

The drug-linker linkage determines the DAR that are related to the efficacy of ADC. Generally, the therapeutic potency of ADC gradually increases *in vitro* with the increase of DAR whereas the therapeutic index *in vivo* decreases (Hamblett et al., 2004), which may due to with the enhancement of DAR accelerates the clearance of ADC which is closely related to the hydrophobicity of ADC (Lyon et al., 2015). The hydrophobicity is determined by the amounts of payloads per antibody and the design of drug-linker (Doronina et al., 2014). It is the main reason for the failure of ADC in the clinical application that the concentration of payload is deficient to treat tumors on account of the DAR of ADC in clinical stage generally control to 3.5–4 (Beck et al., 2017). Thus, augmenting the hydrophilicity of ADC with high DAR by the design of drug-linker exquisitely will improve the efficacy *in vivo* (Pabst et al., 2017). Some hydrophilic groups such as PEG or PHF may improve this dilemma. Accurately connecting these hydrophilic groups to a linker will effectively improve the efficacy of the ADCs. For example, Trastuzumab–PHF–Vinca ADC with DAR of 20 demonstrated a potent anti-tumor activity and decent pharmacokinetic profile due to the high hydrophilicity of PHF (Yurkovetskiy et al., 2015).

At the same time, MDR^+^ tumor cells are insensible to some ADCs due to the fact that many payloads applied to ADCs are hydrophobic, which are the substrates of the MDR1 transporter. By improving the hydrophobicity of the drug-linker, it seems to be able to bypass MDR (Kovtun et al., 2010; Shefet-Carasso and Benhar, 2015).

## Other Parameters Correlated With the Efficacy of Adcs

### The Relationship Between the Internal Environment and Activity of ADCs

Normally, we consider the internalization that influences the efficacy of ADCs to be regulated by antigen. Recently, Lee et al. (2018a) demonstrated that the internalization may be mainly determined by the cellular environment rather than the antigen, which brought another hint that the development of the ADCs has to consider a variety of parameters besides the choice of target and the design of the linker. The characteristics of tumor cells also affect the activity of ADCs, including the endothelium, interstitial, and epithelial barriers which could limit ADCs uptake in the tumor, resulting in a small fraction of the injected dose reaching the desired tumor target (Perez et al., 2014). Intra-tumor distribution of ADCs also affects the anti-tumor efficacy (Tsumura et al., 2018).

Sometimes the efficacy of the ADCs does not have a positive correlation with the dose of the injection of ADCs. In addition to being interfered by the payload concentration threshold, the activity of ADCs could be affected by the saturation of the antigen–antibody combination, which causes the concentration of the ADCs in the circulation to be higher than the concentration of the corresponding receptors (Mager, 2006). Some antigens may shed from the tumor cells and circulate in the blood system to alleviate invalid combination with antibodies, which is also able to enhance the efficacy of ADCs (Pak et al., 2012). These internal factors seem to be imperative considerations when designing ADCs in the future.

### The External Conditions Related to Activity of ADCs

Another point worth attention is the choice of assessment method of safety and efficacy of ADCs. Owing to the ADC subjects to some physical and chemical conditions such as storage conditions, which is able to cause degradation or aggregation of ADCs to influence the assessment of ADCs’ activity (Mohamed et al., 2018). Therefore, the assessment method must be considered to some extent.

## Conclusion and Perspective

With in-depth understandings of antibodies, linkers, and payloads, ADCs have also achieved great development. The linkage strategy and target diversity have already improved the delivery of the payloads to tumor tissues and reduced exposure to normal tissues. With the development of payloads, some novel potent payloads are used by ADCs, which allows researchers to exploit novel linkers to attach the antibody and payloads without disturbing their potency (Dragovich et al., 2018). Furthermore, some irrelevant antigen-target ADCs also may exert toxicity to tumor cells due to the vascular gap of tumors relative to the normal tissues, which is big enough to make ADCs penetrate into tumor cells (Cardillo et al., 2011), indicating the specific recognition of ADCs by tumor tissues on another aspect.

Some prodrug strategies also are used in ADCs design, which modified the toxic payloads to inactive prodrugs, then utilized self-immolation groups and took advantage of the intratumoral environment to reduce the prodrugs to prototype drugs to exert intrinsic activity (Pei et al., 2018). Moreover, nanoparticles combining with the strategy of ACD prodrugs could also increase the activity and circumvent MDR (Qi et al., 2017). The key issues of ADCs are optimization of the appropriate antibody, the choice of proper antigen, the selection of high-activity cytotoxic payloads, stable linkage technology and optimization of DAR in future development. These strategies will improve the efficacy of ADCs that give them a larger market share to replace chemotherapy drugs in medical therapy in the future. At present, ADCs in clinical trials mainly focus on hematological tumors especially Hodgkin lymphoma, because the CD30 is an ideal target, overexpressed in Hodgin lymphoma consistently. With the deep investigation of the target, more ADCs to cure other types of cancer will expand to clinical applications. However, the development of ADCs is costly to make, marked by Adcetris^@^ and Kadcyla^@^ imposing more family burdens on patients.

In recent years, peptide-drug conjugates (PDCs) are also on the stage of targeted-drug conjugation therapy and are considered as part of ADCs. PDCs replace antibodies with peptides, which minimize the molecular weight to alleviate the reduction of tumor cell absorption caused by the larger molecular weight of the ADCs. Also, PDCs could possess better homogeneity due to the few of the attached sites of the peptides. The cost-effectiveness of PDCs is critical to alleviate the pressure on patients during treatment. However, PDCs also have some weaknesses that need to be improved. The vulnerability of PDCs in the blood system is a non-negligible obstacle, but it is difficult to improve half-life and reduce off-target toxicity by modifying structure of PDCs without destroying activity. Therefore, we must master more comprehensive knowledge to improve ADCs or PDCs. Whether used alone or in combination with other therapies, the toxicity of ADCs and PDCs must be better understood to adjust the therapeutic index based on the minimum effective dose of the drug in tumor cells and the maximum tolerated dose for normal tissues.

## Author Contributions

HT wrote the review manuscript. YL, ZY, MS, and WL modified the review. LL and QH edited the review including grammar. The correspondent authors MW and YJ provided the thought.

## Conflict of Interest Statement

The authors declare that the research was conducted in the absence of any commercial or financial relationships that could be construed as a potential conflict of interest.

## Footnotes

1 https://clinicaltrials.gov

## Supplementary Material

TABLE S1 The ADCs of cancer therapy in clinical development.

## Abbreviations

ADCs: antibody–drug conjugates
ALCL: anaplastic large cell lymphoma
AML: acute myeloid leukemia
DAR: drug-to-antibody ratio
MDR: multiple drug resistance
MMAE: monomethyl auristatin E
PDBs: pyrrolobenzodiazepine dimers
PEG: polyethylene glycol
PHF: hydroxymethyl-formal
SMCC: succinimidyl-4-(*N*-maleimidomethyl)-cyclohexane-1-carboxylate
SMCC-DM1: succinimidyl 4-(*N*-maleimidomethyl) cyclohexane-1-carboxylate-maytansinoid
sulfo-SPDB-DM4: *N*-succinimidyl-4-(2-pyridyldithio)-2-sulfo butanoate-maytansinoid
val-cit-PABC-MMAF: valine-citrulline-p-aminobenzyl-carbamate-monomethyl auristatin F.
